# Is Aducanumab for LMICs? Promises and Challenges

**DOI:** 10.3390/brainsci11111547

**Published:** 2021-11-22

**Authors:** Illangage P. C. Gunawardena, Thaarvena Retinasamy, Mohd. Farooq Shaikh

**Affiliations:** 1 Clinical School Johor Bahru, Jeffrey Cheah School of Medicine and Health Sciences, Monash University Malaysia, Johor Bahru 80100, Johor, Malaysia; igun0005@student.monash.edu; 2 Neuropharmacology Research Strength, Jeffrey Cheah School of Medicine and Health Sciences, Monash University Malaysia, Bandar Sunway 47500, Selangor, Malaysia; thaarvena.retinasamy@monash.edu

**Keywords:** Alzheimer’s disease, aducanumab, LMICs, APOE, burden of disease, treatment cost

## Abstract

Aducanumab, a human monoclonal antibody, was approved in June of 2021 as the first disease-modifying treatment for Alzheimer’s disease by the United States Food and Drug Administration (U.S. FDA). A substantial proportion of patients with Alzheimer’s disease live in low- and middle-income countries (LMICs), and the debilitating effects of this disease exerts burdens on patients and caregivers in addition to the significant economic strains many nations bear. While the advantages of a disease-modifying therapy are clear in delaying the progression of disease to improve patient outcomes, aducanumab’s approval by the U.S. FDA was met with controversy for a plethora of reasons. This paper will provide precursory insights into aducanumab’s role, appropriateness, and cost-effectiveness in low- and middle-income countries. We extend some of the controversies associated with aducanumab, including the contradicting evidence from the two trials (EMERGE and ENGAGE) and the resources required to deliver the treatment safely and effectively to patients, among other key considerations.

## 1. Introduction

Alzheimer’s disease (AD) is an irreversible neurodegenerative disorder that is characterised by the progressive deterioration of certain parts of the brain that are essential for learning and memory. The disease progresses in stages over months or years, gradually affecting memory, reasoning, judgement, language, and eventually even simple tasks [1]. AD was first described by a German psychiatrist, Alois Alzheimer, in 1906, while performing an autopsy on a woman with memory and language impairment, where abnormal structures called senile plaques and neurofibrillary tangles were found throughout the cerebral cortex of her brain [2]. The clinical expression of AD is believed to begin decades before the onset of the disease, which is observed via the formation of specific AD pathology, amyloid-beta (Aβ) plaques between neurons, and the accumulation of intracellular neurofibrillary tangles composed of tau. These AD pathological hallmarks then trigger neuronal dysfunction, neurotoxicity, and inflammation, leading to cognitive dysfunction [3]. During the initial stages of the disease, neuronal and synaptic impairment occurs within the para-hippocampal regions, which are the regions of the brain responsible for the formation of new memories. However, as the disease progresses, the neuropathology continues to spread, triggering cortical atrophy and ventricular enlargement, which causes the total brain mass to reduce by up to 35% [4].

Among causes of dementia, such as cerebrovascular disease, Lewy body disease, and frontotemporal lobar degeneration (FTLD), AD is the most common cause of dementia worldwide [5]. AD accounts for 60–70% of all dementia cases, affecting at least 27 million people globally [6]. The incidence of AD increases exponentially with age [7]. Evidence suggests that dementias, including AD, are more prevalent among women than men [8,9]. It is often challenging to clinically distinguish AD from other types of dementia. The disease is usually difficult to diagnose until post-mortem, where Aβ plaques and tau neurofibrillary tangles within the brain are identified. Thus, a diagnosis is only feasible based on the symptoms and cognitive assessments, thereby making diagnosing, treating, and managing AD extremely demanding and, consequently, making analysing the burden of disease by dementia subtype challenging. This may also explain the scarcity of epidemiological data in low- and middle-income countries (LMICs).

Aducanumab is a human monoclonal antibody that works to reduce Aβ load in the brain; it is the first disease-modifying therapy to be approved for AD treatment [10]. On 7 June 2021, the United States Food and Drug Administration (U.S. FDA) approved aducanumab via an accelerated approval pathway [11]. Current AD treatment is centered on supportive care to manage the debilitating symptoms of dementia, and pharmacotherapy goals of mainstay classes of drugs, such as cholinesterase inhibitors (ChEIs) and N-methyl-D-aspartate (NMDA) receptor antagonists, do not modify the course of the disease. For this reason, there was much enthusiasm by those impacted by AD, including patients, caregivers, clinicians, and the broader community.

Nonetheless, this was overshadowed by controversy surrounding aducanumab’s efficacy and performance in the two trials, namely EMERGE and ENGAGE, as well as questions of adverse events, the need for constant monitoring, and its high cost. Although aducanumab is currently new to the market, it is vital to gauge the suitability of this drug in the context of LMICs for the treatment of AD by taking a multifactorial approach. It is estimated that approximately half of the patient population with dementia (including all subtypes) live in LMICs [12]. LMICs have an increasingly ageing population, characterised by a demographic transition from a high to low shift in mortality and fertility rates. As a result, many LMICs face an increasing burden due to chronic, non-communicable diseases (NCDs) proportional to this epidemiological shift [13]; an increase in the number of AD cases in these countries is likely to be observed. This necessitates the need for a disease-modifying therapy, such as aducanumab.

## 2. Management of Alzheimer’s Disease

Before the approval of aducanumab, a disease-modifying therapy, the mainstream treatment goals were improving cognitive and functional deficits, reducing behavioral disturbances, and ultimately improving patients’ quality of life [14]. This was achieved using ChEIs, NMDA receptor antagonists, and SSRIs (selective serotonin-reuptake inhibitors) or SNRIs (serotonin-noradrenaline reuptake inhibitors). Most drugs currently in development at various stages of clinical trials are disease-modifying therapies; in fact, out of 126 agents in development in 2021, 104 (82.5% of all agents in trial) are disease-modifying agents [15]. A further 16 of these drugs target amyloid pathology specifically. Other disease-modifying therapies are centered on mechanisms based on synaptic plasticity and neuroprotection, tau pathology, oxidative stress, vasculature, and inflammation, to name some. The other non-disease modifying agents aim for the treatment of neuropsychiatric and behavioral symptoms associated with AD. For example, Sigma-1 receptor agonists, NMDA receptor antagonists, and Alpha-1 antagonists are being studied in various trials [15]. The following discussion will focus on drugs which are currently available on the market for the treatment of AD.

### 2.1. ChEIs and NMDA Receptor Antagonists

Two major classes of drugs, namely ChEIs and NMDA receptor antagonists, are currently used for improving symptoms and patient outcomes. ChEIs (donepezil, galantamine, or rivastigmine) enhance cortical cholinergic transmission and function by inhibiting synaptic cleft cholinesterase [16]. The benefit of symptomatic relief is modest; oral rivastigmine and oral galantamine are used in mild to moderate AD, while oral donepezil and transdermal rivastigmine are used in mild to severe AD [16,17]. NMDA receptor antagonists (memantine) work by blocking excessive, pathological NMDA receptor stimulation, and it is thought to be neuroprotective [18]. While memantine appears to have modest benefits in patients with moderate, severe, and advanced disease, especially when combined with a ChEI, there is limited evidence for its use in mild AD (although it is widely used off-label) [18].

### 2.2. SSRIs and SNRIs

Behavioral and psychological symptoms of AD are wide-ranging, from insomnia, anxiety, agitation, and apathy to depression, delusions, and hallucinations [19]. SSRIs, like sertraline, citalopram, and escitalopram, are preferred in depressed patients with AD, while atypical antidepressants and SNRIs may also be used [20]. Other pharmacological agents include low-dose trazodone or orexin antagonists for improved sleep. Trazodone and SSRIs may be considered for agitated behaviors, while risperidone, an atypical antipsychotic, may reduce general neurobehavioral symptoms in AD patients [14].

### 2.3. Aducanumab—Antibody-Based Immunotherapy

**Figure 1:**
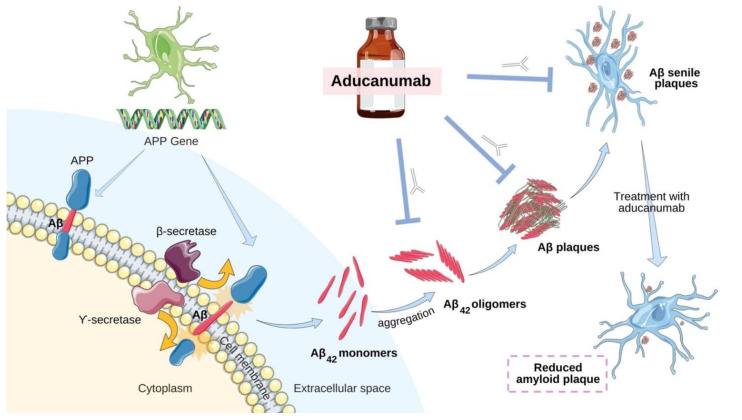
Aβ aggregation pathway and mechanism of action of aducanumab. APP is cleaved by β-secretase and γ-secretase to form Aβ_42_ monomers, oligomers, and eventually senile plaques (note that protofibrils and fibrils are not illustrated in this figure); these are targets for clearance by aducanumab resulting in reduced amyloid burden. Parts of the figure were illustrated using images from Servier Medical Art by Servier, licensed under a Creative Commons Attribution 3.0 Unported License (https://creativecommons.org/licenses/by/3.0/ (accessed on 10 November 2021)).

Aducanumab, which is marketed as ADUHELM, is AD’s first targeted therapy, and its mechanism of action is based on the amyloid hypothesis. The amyloid hypothesis has been the mainstream concept underlying AD research for many years. It has been postulated that the misfolding and aggregation of Aβ peptides as senile plaques in the brain causes neurodegeneration [21]. Additionally, Aβ aggregation has also been said to be involved in triggering other closely related pathophysiological pathways, like tau hyperphosphorylation, inflammation, oxidative stress, and generation of reactive oxygen species (ROS), among others, which ultimately lead to neuronal toxicity and cell death [22]. Aβ peptides are obtained from the amyloid precursor protein (APP) via the action of secretases, which are protease enzymes. In normal physiological conditions, APP is usually cleaved by both α-secretase and γ-secretase, which produce non-toxic fragments that are cleared from the brain. However, under extreme pathophysiological conditions, the APP are cleaved by β-secretase and γ-secretase instead, producing Aβ peptide fragments, particularly Aβ_42_, which are highly amyloidogenic. These fragments then proceed to misfold, forming toxic oligomers, protofibrils, fibrils, and senile plaques in extracellular regions of the brain (Figure 1) [22]. The resultant Aβ oligomers interfere with the signalling cascade in the synaptic cleft, ultimately causing synaptic dysfunction and neuronal death.

Aducanumab uses an antibody-based immunotherapeutic approach by choosing human B-cell clones-activated Aβ aggregates epitopes [23]. When various human memory B cells were selected for testing reactivity against aggregated Aβ, aducanumab, a human monoclonal antibody, was found to be selectively reactive with Aβ aggregates, soluble oligomers, as well as insoluble fibrils; hence, it was selected as the lead drug candidate [24]. Aducanumab was found to cross the blood-brain barrier in preclinical studies, which further reduced brain amyloid burden [23]. Preclinical studies using Tg2576 mice reported that intraperitoneally injected aducanumab bound and aided in the clearance of parenchymal plaques, preventing micro hemorrhages [25]. Additionally, the accumulation of brain macrophages around the remaining plaques was also reported, thus implying the possibility of phagocytosis being used to remove the Aβ plaques, potentially slowing neurodegeneration and disease progression [26]. Aducanumab has since been deemed as the first approved treatment to treat AD’s root cause instead of just treating the symptoms.

## 3. Aducanumab in Low- and Middle-Income Countries

AD places burdens on an individual level, affecting patients, caregivers, and families while also burdening and straining healthcare systems at a societal level. In terms of years of life lived with disability (YLD) due to non-communicable disease, dementia (including AD) accounts for 11.9% of the total number of years [7]. In 2019, AD and all dementias accounted for 5.6% of all global disability-adjusted life years (DALYs) and was the fourth-highest cause of DALYs in patients aged 75 and older [27]. There is a demand for disease-modifying AD therapies, as an increase in the number of dementia and AD cases, especially in LMICs, would exert an unwarranted burden on patients and caregivers. At the same time, resource limitations would negatively impact healthcare systems in these countries. There are currently no biosimilars or generic equivalents of aducanumab. It is the only disease-modifying AD therapy currently in the market, targeting the underlying pathophysiology of the AD disease process [28,29,30]. The economic cost of dementia (including AD) increased by 35.4% from U.S. $604 billion in 2010 to U.S. $818 billion in 2015, which is 1.09% of the world’s GDP [12,31]. An estimated U.S. $715.1 billion or 86% of these costs were from high-income countries (HICs), while LMICs accounted for a sum of U.S. $102.8 billion. A disease-modifying therapy could potentially decrease the burden of AD in terms of mortality and morbidity and improve health outcomes by generating an enduring clinical effect.

Comparing the efficacy of aducanumab directly with more conventional drug classes, such as ChEIs and NMDA receptor antagonists, is challenging, as the pharmacotherapy goals are inherently different. Aducanumab is a recombinant monoclonal antibody based on the principles of passive immunotherapy. It works by selectively binding Aβ fibrils and soluble oligomers, reducing amyloid-beta dose- and time-dependently [32]. ChEIs and NMDA receptor antagonists, on the other hand, aim for symptomatic alleviation. Evidence shows that donepezil, rivastigmine, and galantamine yields modest improvements in cognitive and clinical function in patients with mild to moderate AD in the short and long term [33]. Table 1 represents a crude comparison of the various characteristics of aducanumab, ChEIs, and NMDA receptor antagonists, including pharmacotherapy goals, mechanism of action, and efficacy, among others. The efficacy data are based on the most common primary outcome measures of cognitive function—with aducanumab’s data based on the randomised-control trial (EMERGE), while meta-analyses were used for the remaining drug classes. Care should be taken when interpreting and comparing efficacy between the drugs, as it is subject to variabilities in study design, including patient characteristics and indications. This is due to a lack of head-to-head clinical trials between aducanumab and other drugs currently available on the market; hence, the data in Table 1 that directly compares the efficacy of different drug classes serves an exploratory purpose. Commenting on the robustness of the data presented, the quality of evidence was moderate in studies used in the meta-analyses for donepezil [34] and rivastigmine [35]. There was considerable heterogeneity in some outcome measures in the galantamine [36] and memantine [37] meta-analyses. All meta-analyses presented were subject to publication bias, and most studies included in the analyses were industry-funded.

### 3.1. Accelerated Approval and the Efficacy of Aducanumab

**Figure 2:**
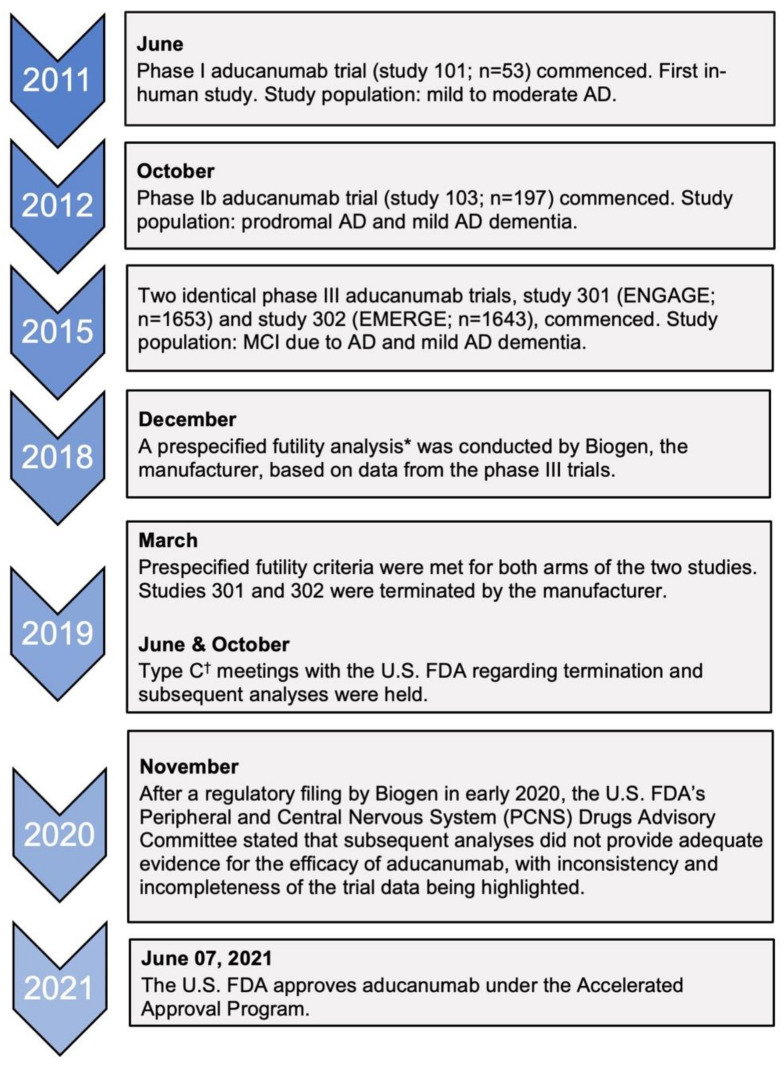
Timeline of clinical trials of aducanumab and key regulatory decisions. AD, Alzheimer’s disease; MCI, mild cognitive impairment. * This was defined as the conditional power being less than 20% for both dose arms of the trials to meet the primary endpoint [40]. ^†^ Type C meetings are those that do not fall into type A meetings (due to stalled product development) or type B meetings (for example, after a trial’s end-of-phase) [42].

The first aducanumab trials started in 2011 with a phase I study (study 101) after pre-clinical trials with transgenic mice showed reduced amyloid burden in the brain [25,39]. The clinical trials that are important in assessing the efficacy of aducanumab are studies 103 (phase Ib) and the two identical phase III trials: 301 (ENGAGE) and 302 (EMERGE) [40]. Figure 2 is a timeline that summarizes the key events leading up to the accelerated approval of aducanumab.

Of the two phase III clinical trials, only the high-dose arm of one trial, EMERGE, met its primary endpoint by demonstrating improvements in the Clinical Dementia-Sum of Boxes (CDR-SB) score in addition to showing benefits in other secondary outcomes, such as the MMSE score, ADAS-Cog-13, and ADCS-ADL-MCI scores [40]. However, the low-dose arm did not reveal any benefit of aducanumab compared to the placebo, and no benefits were observed in either arm of the ENGAGE trial [29]. In fact, in the ENGAGE trial, it was noted that the CDR-SB score change in the high-dose arm was quantitatively worse than placebo at 78 weeks [40]. Prior to the current analysis by the manufacturer, the two trials were halted in March 2019 after a planned interim analysis met the criteria for futility [41]. On that account, confidence in the efficacy of aducanumab would need to be tempered due to the contradicting evidence presented from the two trials.

Furthermore, the efficacy of aducanumab was determined in research settings, and therefore, clinical practice may vary. The placebo-controlled EMERGE and ENGAGE trial’s population included individuals with early AD (i.e., those with mild cognitive impairment due to AD or those with mild AD) [29]. Moderate to severe AD patients make up approximately 50% of the total number of individuals living with AD [43]. The suitability and efficacy of aducanumab were not assessed in these patient groups, effectively limiting access to half of all patients living with AD, which will continue to contribute significantly to the burden of disease. Additionally, there is an intense debate surrounding the clinical significance observed in the EMERGE trial, as the hypothesis that clearance of Aβ protein equates to clinical improvement is inconclusive and is yet to be demonstrated [14]. As the trials have utilized a surrogate endpoint that facilitated the U.S. FDA’s accelerated approval, the data can only predict a clinical outcome for the treatment of AD. As a result, full approval depends on a phase IV confirmatory trial, which aims to measure the clinical benefit [44]. Consequently, aducanumab’s applicability to a limited subset of AD patients and its currently contended effectiveness would negatively affect the suitability of this drug in many countries, including in LMICs.

### 3.2. Treatment Challenges

An issue that would impact the suitability of aducanumab in many LMICs is the complexity of the treatment regimen and the need for robust healthcare systems to deliver therapies to patients. Aducanumab is administered by intravenous infusion every four weeks, with increasing titrations every two weeks for the first 32 weeks, followed by a constant high dose beyond week 36, with each infusion lasting 1 h [40]. Additionally, ascertaining the amyloid burden in patients before initiating treatment is crucial to guide clinical diagnosis and to assess the suitability of aducanumab. This is achieved by positron emission tomography (PET) for a visual read or through an invasive cerebrospinal fluid (CSF) quantitative analysis [10]. Amyloid-PET scans should be interpreted cautiously by trained radiologists and nuclear medicine specialists [10]. In addition to resource limitations and healthcare availability and accessibility, this complex treatment regimen highlights the importance of continuity and coordination in healthcare, which is associated with improved health outcomes [45]; however, such measures are lacking in many LMICs [46]. Safety and competency among healthcare workers are essential in delivering novel therapies effectively; evidence suggests that healthcare service competence and safety are deficient in LMICs [45]. The number of physicians per 1000 people in high-income countries was 3.1, while it was 1.3 in low- and middle-income countries [47], further emphasising resource limitations. On the contrary, a prospective advantage of aducanumab’s approval is that it could lay the foundation for future therapies for AD in terms of advancing and improving treatment delivery to patients, which can improve health outcomes. For instance, long-term data from follow-up trials would be crucial in determining efficacy as well as providing an opportunity for head-to-head trials with existing therapies to be conducted. Due to the nature of AD and its multifaceted pathophysiology, combination therapy involving multiple targets may be necessary [48]. Therefore, aducanumab could serve as a catalyst towards better AD treatment in the future. Nonetheless, considering the importance of care continuity, follow-ups, and the general complexities associated with dosing intervals, which are all augmented by a general absence of high-quality healthcare coverage, introducing aducanumab to LMICs would be challenging.

### 3.3. Adverse Effects

Adequate follow-ups during the treatment phase are crucial for monitoring severe adverse events. ARIA (amyloid-related imaging abnormalities) due to oedema (ARIA-E) and brain microhemorrhage or localised superficial siderosis (ARIA-H) were frequently seen in the treatment groups [40]. In the high-dose treatment arm of the EMERGE and ENGAGE phase III trials, 41.3% of individuals experienced ARIAs compared to 10.3% in the placebo arm [40]. In addition to a pre-treatment MRI, frequent scans were performed to monitor the ARIAs of aducanumab. Individuals were scheduled to have five MRI scans of the brain in the first year of treatment alone, followed by two more scans in the last six months of treatment [40]. In clinical practice, this would mean a pre-treatment MRI, followed by two more brain MRI scans before the seventh and twelfth doses, which is in addition to more scans if patients experience symptoms related to ARIAs. To put it into perspective, in high-income countries, the number of MRI scanners per million inhabitants is 27.3, which contrasts with 3.4 scanners and 0.4 scanners per million inhabitants in upper-middle-income and lower-middle-income countries, respectively, and 0.2 scanners per million inhabitants in low-income countries [49]. If such monitoring practices cannot be implemented effectively in these countries, patients with severe ARIAs would not be identified and managed early, compounding the burden on patients, caregivers, healthcare systems, and economies.

### 3.4. Apolipoprotein E and Interethnic Differences

The synthesis, clearance, and accumulation of Aβ are influenced by a variety of factors. In the less common familial AD, mutations in the APP gene or PSEN1 gene may lead to increased Aβ accumulation [50]. Age-related processes, including neuronal stress, microglia-related inflammation, and a negative impact on protein homeostasis, may affect Aβ aggregation [51,52,53]. Other factors, such as insulin-like growth factor (IGF) resistance and diabetes, traumatic brain injuries, and the human microbiota, have also been studied [50]. However, for sporadic AD, which is more common, the presence of the ε4 allele of apolipoprotein E (APOEε4) is implicated, while BIN1 (bridging integrator-1) and TREM2 (Triggering Receptor Expressed On Myeloid Cells 2) also play a role [50]. Apolipoproteins usually aid in the transport of lipids in the body; however, APOE4 can also form stable complexes with Aβ, impacting its clearance from the brain. Therefore, APOEε4 genotype carriers have a higher amyloid load than non-carriers, and amyloid positivity is associated with greater cognitive impairment [54,55].

**Figure 3:**
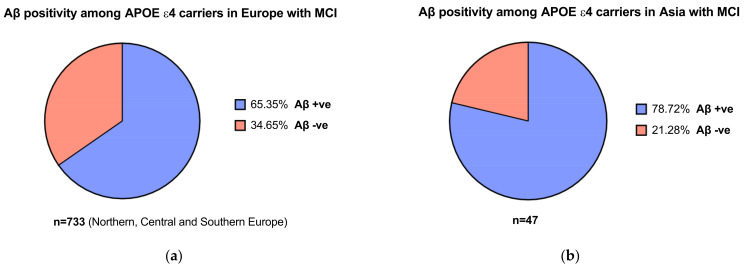
(**a**) Aβ positivity among APOEε4 carriers with mild cognitive impairment (MCI) in Europe (**b**) and in Asia. Data obtained from Mattson et al. [57].

In the EMERGE and ENGAGE trials, participants underwent genetic screening for the presence of the APOEε4 allele [30]. It was noted that 65–75% of patients with AD carry the APOEε4 allele [56]. For instance, Mattsson and colleagues’ [57] meta-analysis found that the frequency of APOEε4 carriers in patients with AD was 68.9% in Northern Europe and 52.1% in Asian populations. For patients with MCI, this was 52.5% vs. 33.3% in Northern Europe vs. Asia. The proportion of APOEε4 carriers with MCI who were Aβ positive in both Asian and European populations were significant compared to patients who were Aβ negative (Figure 3a,b) [57]. This is important, as the population assessed in the aducanumab trials were patients with confirmed amyloid pathology [10], which relates to the mechanism of action of the therapeutic. While more studies need to be conducted to confirm differences in APOEε4 allele frequencies across different populations, it may indicate the relative prevalence of amyloid pathology across different regions and ethnicities and, by extension, may guide cost-effectiveness assessments in nations including in LMICs.

**Figure 4:**
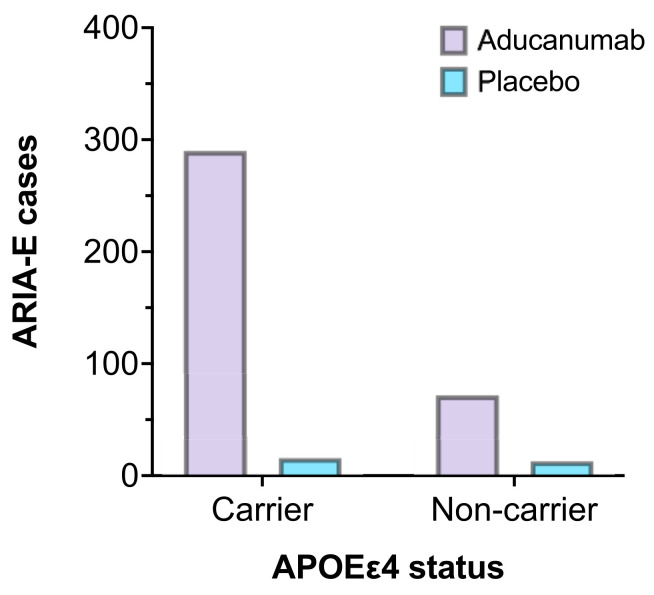
The number of ARIA-E cases by APOEε4 carrier status in EMERGE and ENGAGE (aducanumab 10 mg/kg, n = 362 vs. placebo, n = 29) [44].

Most notably, APOEε4 status was associated with an increased incidence of ARIA-E. As a result, dosing was adjusted in the initial stages of the trials to allow for lower doses in participants who carried the gene [29]. Figure 4 represents the number of participants who experienced ARIA-E in EMERGE and ENGAGE, stratified by APOEε4 carrier status compared to placebo. Furthermore, homozygous carriers may be predisposed to more frequent and severe ARIA than those who carry only one copy of the allele [58]. Although genotype testing is currently not indicated as part of the pre-treatment procedure, clinicians must carefully assess the risk-benefit balance. Additionally, if such risk-stratification measures are required as part of a full regulatory approval in the future, it would only serve to compound resource and economic burdens when delivering this complex therapy to patients.

The ethno-racial differences in AD pathology could impact the cost-effectiveness of aducanumab in LMICs. In diagnostics, P-tau biomarkers (p-tau181 and p-tau217) were associated with Aβ pathology on PET [59]. Brickman et al. [59] found that concentrations of p-tau biomarkers did not differ across Non-Hispanic Whites, Black people, and Hispanics. However, another study found that Black people had lower CSF phosphorylated-tau (p-tau181) and total tau (t-tau) levels when compared to Caucasians, independent of cognition [60]. Although there is no clear association between the prevalence of amyloid pathology and socioeconomic status, and due to limitations in the literature concerning the interethnic impact of both allele frequencies and biomarker levels on amyloid pathology, aducanumab’s use in these populations must be assessed to further clarify its cost-effectiveness, especially in the context of LMICs.

### 3.5. The Economic Burden

As announced by the manufacturer, aducanumab’s wholesale cost is U.S. $56,000 annually per patient [30]. The Institute for Clinical and Economic Review’s (ICER) value-based analysis suggests a significantly lower price between U.S. $2500 and U.S. $8300 per annum, which is comparable to existing classes of drugs [30] (Table 2). The prevalence of AD is significant; however, its relatively low incidence compared to other non-communicable diseases could mean lower total public health expenditure. Nevertheless, ICER’s report estimated the annual United States budget impact to be U.S. $819 million for aducanumab [30]. There is a pronounced incongruity between pharmaceutical spending in high-income countries and LMICs—while 84% of the global population reside in LMICs, it only accounted for 21.5% of the total global pharmaceutical expenditure [61]. Since aducanumab targets the early AD stage to prolong disease progression [40], healthcare systems would need to factor in the costs of managing patients over a longer period. Training individuals regarding safe administration, monitoring, follow-ups, and best practices would incur high costs. Moreover, the costs associated with pre-and post-treatment follow-ups, such as the need for multiple MRI scans of the brain, as described, are likely to burden national healthcare systems and economies while serving as a barrier by making healthcare inaccessible to patients. Conversely, the epidemiological shifts seen in LMICs towards lower mortality rates [13] may reflect improving economies, which could indicate a better level of economic sustainability for drugs such as aducanumab. Nevertheless, determining and comparing the cost-effectiveness of aducanumab between countries is challenging due to the different architectures of healthcare systems, inconsistent funding, utilisation of resources, and the individual nature of policy and legislation development.

## 4. Conclusions

In summary, disease-modifying treatment for Alzheimer’s disease, such as aducanumab, has the potential to ease the burden at both an individual and societal level, and such an intervention would be highly beneficial in many countries. However, we have identified areas of concern with regards to the suitability of aducanumab in LMICs, including reservations about its clinical efficacy, the complexity associated with the safe delivery of aducanumab to patients, challenges with follow-ups and monitoring, the relative prevalence of apolipoprotein E in different regions, and most prominently, its high cost. These factors need to be considered when evaluating the cost-effectiveness of aducanumab in LMICs. By extension, these concerns could be applicable to future therapeutics, especially antibody-based immunotherapies, and highlights the need for more accessible options in the context of LMICs to reduce the burden of disease in these nations.

## Figures and Tables

**Table 1:** Direct comparison between aducanumab and mainstay pharmacotherapy of AD.

	Monoclonal Antibodies	Cholinesterase Inhibitors (ChEIs)	N-methyl-D-aspartate (NMDA) Receptor Antagonists
**Drug**	Aducanumab	Donepezil, rivastigmine, and galantamine	Memantine
**Pharmacotherapy goal(s)**	Disease-modifying treatment to reduce cognitive decline.	Symptomatic management of cognition and global functioning.	Symptomatic management of cognition and global functioning.
**Mechanism of action**	Selectively targets and clears Aβ aggregates, Aβ fibrils, and soluble oligomers. A reduction in Aβ build up in the brain is expected to demonstrate a reduction in cognitive decline in patients [23].	Increases cholinergic transmission by inhibiting cholinesterase at the synaptic cleft, thereby improving cortical cholinergic function [33].	Exerts neuroprotective effects by blocking pathological stimulation of glutamate NMDA receptors, thereby decreasing excitotoxicity [33].
**Indication**	Mild cognitive impairment (MCI), mild AD [10]	Mild to moderate AD, advanced disease [35]	Moderate to severe AD, mild AD (off-label) [18]
**Route of administration**	Intravenous infusion	Oral, transdermal patch (rivastigmine only)	Oral
**Efficacy in terms of cognitive function**	A statistically significant improvement in various scales of cognitive function was observed in the high-dose arm of EMERGE [29].	A meta-analysis of ChEIs revealed modest improvements [38].	A reduction in deterioration on different scales of clinical efficacy compared to placebo was observed in patients with moderate to severe AD [18].
	Difference vs. placebo: −0.39 (95% CI 0.69 to −0.09) [29] Score: CDR-SB *	**Donepezil**	MD −1.02, (95% CI −1.66 to −0.39, *p* = 0.002) [37] Scale: ADAS-Cog ^†^
		MD −2.67, (95% CI −3.31 to −2.02) [34] Scale: ADAS-Cog ^†^	
		MD 1.05, (95% CI 0.73 to 1.37) [34] Score: MMSE ^‡^	
	Difference vs. placebo: −1.400 (*p* = 0.00967) [29] Scale: ADAS-Cog **	**Galantamine**	
		MD −2.95, (95% CI −3.32, −2.57) [36] Scale: ADAS-Cog ^†^	
		MD 2.50, (95% CI 0.86 to 4.15) [36] Score: MMSE ^‡^	
	Difference vs. placebo: 0.6 (*p* = 0.05) [29] Score: MMSE ^‡^	**Rivastigmine**	
		MD −1.79, (95% CI −2.21 to −1.37) [35] Scale: ADAS-Cog ^†^	
		MD 0.74, (95% CI 0.52 to 0.97) [35] Score: MMSE ^‡^	

AD, Alzheimer’s disease; Aβ, amyloid-beta protein; ADAS-Cog, Alzheimer’s Disease Assessment Scale-Cognitive Subscale; CDR-SB, Clinical Dementia Rating-Sum of Boxes; CI, confidence interval; MD, mean difference; MMSE, Mini-Mental State Exam; SMD, standard mean difference. * CDR-SB scores range from 0 to 18; higher scores mean greater disease severity. ^†^ ADAS-Cog scores range from 0 to 70; higher scores mean greater cognitive impairment. ** The aducanumab trials utilised the ADAS-Cog-13 scale; scores range from 0 to 85; higher scores mean greater cognitive impairment. ^‡^ MMSE scores range from 0 to 30; higher scores mean less cognitive impairment.

**Table 2:** Additional differences between aducanumab and conventional drugs for the treatment of AD.

	Monoclonal Antibodies	Cholinesterase Inhibitors (ChEIs)	N-methyl-D-aspartate (NMDA) Receptor Antagonists
**Drug**	Aducanumab	Donepezil, rivastigmine, and galantamine	Memantine
**Functional outcomes**	MD 1.70 (95% CI 0.71 to 2.69) (high-dose arm of EMERGE) [29] Scale: ADCS-AD ^a^	Donepezil: SMD 0.22 (95% CI 0.12–0.33) [34]	MD 0.95 (95% CI 0.22 to 1.76) [18] Scale: ADCS-AD ^c^
		Galantamine: SMD 0.19 (95% CI 0.01–0.37) [33]	
		Rivastigmine: MD 1.80 (95% CI 0.20 to 3.40) [62]	
		Scale: ADCS-AD ^b^	
**Entry to institutional or nursing care**	Not assessed	No significant benefit in terms of delay of entry to institutional care [63].	No effect on the rate of nursing home placement [64].
**Adverse effects**	ARIA-E and ARIA-H Symptoms: headaches, confusion, dizziness, falls, vision changes, and nausea [40].	Donepezil: gastrointestinal symptoms (upset stomach, nausea, diarrhoea, and anorexia)	Dizziness, confusion, weight gain, hallucinations [18].
		Galantamine: gastrointestinal symptoms	
		Rivastigmine (patch): nausea, vomiting, anorexia, headaches, dizziness	
		General vagotonic symptoms: bradycardia and hypotension [38].	
**APOE genotyping**	Not a requirement. However, genetic screening may help ascertain ARIA risk.	Not required	Not required
**Pre-treatment amyloid status**	Amyloid-PET or CSF analysis may be conducted [10].	Not applicable	Not applicable
**Baseline MRI scan of the brain**	Required (a recent scan within one year prior to initiating therapy) [10].	For clinical diagnosis	For clinical diagnosis
**Follow-up MRI scans of the brain**	Two scans prior to the seventh and twelfth doses. Additional monitoring of ARIAs with MRI if symptomatic [10].	Not required	Not required
**Average annual cost (U.S. $)**	56,000 *	2796 ^†^	4096 ^‡^
**Generics or biosimilars**	None	Available	Available

AD, Alzheimer’s disease; Aβ, amyloid-beta protein; ADCS-ADL, Alzheimer’s Disease Cooperative Study-Activities of Daily Living Inventory; amyloid-PET, amyloid-positron emission tomography; ARIA-E, amyloid-related imaging abnormalities due to oedema; ARIA-H,amyloid-related imaging abnormalities due to brain microhemorrhage or localised superficial siderosis; CDR-SB, Clinical Dementia Rating-Sum of Boxes; MMSE, Mini-Mental State Exam; MD, mean difference; SMD, standard mean difference. ^a^ ADCS-ADL-MCI (aducanumab), adapted for patients with mild cognitive impairment (MCI), scored from 0 to 53; higher scores represent greater cognitive impairment. ^b^ ADCS-ADL (donepezil, rivastigmine and galantamine), scored from 0 to 78; higher scores represent greater cognitive impairment. ^c^ ADCS-ADL_19_ (memantine), adapted for patients with severe AD, scored from 0 to 54; higher scores represent greater cognitive impairment. * Cost of the drug only, as set by the manufacturer [30]. Excludes other treatment-related costs. ^†^ Average retail price in the United States of America for one ChEI based on typical dosing in 2012 [65]. ^‡^ Average retail price in the United States of America based on typical dosing in 2012 [61].

## Data Availability

Not applicable.
